# Effects of Shikonin on the Functions of Myeloid Dendritic Cells in a Mouse Model of Severe Aplastic Anemia

**DOI:** 10.1155/2020/9025705

**Published:** 2020-02-20

**Authors:** Mengying Zheng, Bingnan Liu, Yuanyuan Shao, Luogang Hua, Rong Fu, Huaquan Wang, Ting Wang, Weiwei Qi, Zonghong Shao, Chunyan Liu

**Affiliations:** The Department of Hematology, General Hospital of Tianjin Medical University, Tianjin, China

## Abstract

This study is aimed at investigating the effects of shikonin, a pyruvate kinase M2 (PKM2) inhibitor, on the functions of myeloid dendritic cells (mDCs) in a mouse model of severe aplastic anemia (AA) generated by total body irradiation and lymphocyte infusion. Flow cytometry and qPCR were used to determine the proportions of PKM2+ mDCs and other immune indicators in the AA mice. Glucose consumption level, pyruvate generation level, and ATP content were used to determine the level of glycolytic metabolism in the mDCs. The survival rates of AA mice were evaluated after the administration of shikonin or the immunosuppressive agent cyclosporin A. The AA mice displayed pancytopenia, decreased CD4+/CD8+ cell ratio, increased perforin and granzyme levels in CD8+ cells, increased costimulatory CD80 and CD86 expressions, and inadequate regulatory T cell number. *In vivo* animal experiments showed that the shikonin-mediated inhibition of the PKM2 expression in mice was associated with high survival rates. In addition, the administration of cyclosporin A or shikonin decreased the expression of cytotoxic molecules and costimulatory CD80 and CD86 on CD8+ cells. Taken together, the results of this study indicated that shikonin could inhibit the activation and proliferation of mDCs as well as the activation of downstream cytotoxic T cells by reducing the PKM2 level in mDCs.

## 1. Introduction

Severe aplastic anemia (SAA) is a class of highly heterogeneous hematological diseases with complex etiology and pathogenesis [1]. Its clinical symptoms are often characterized by fatal anemia, bleeding, and infection. Immunosuppressive therapy with antithymocyte globulin and cyclosporin A (CsA) is considered a standard treatment approach for SAA patients and is known to improve prognosis. SAA patients exhibit abnormally high levels of activated myeloid dendritic cells (mDCs) that secrete inflammatory factors involved in the generation and activation of T cells. This overactivation of mDCs and T lymphocytes leads to the failure of bone marrow hematopoietic function [2, 3].

Shikonin is an active ingredient in the commonly used Chinese medicinal herb *Lithospermum erythrorhizon* and has been receiving increased attention as a new pyruvate kinase M2 (PKM2) inhibitor [4–6]. Because of its highly selective inhibitory effect on PKM2 instead of PKM1 and pyruvate kinase-L, shikonin is considered the most potent and specific inhibitor of PKM2 identified to date [7]. Recently, the anticancer role of shikonin has also attracted widespread research attention. Studies have shown that shikonin exerts anticancer effects by inhibiting the proliferation of different cancer cells and inducing their apoptosis and autophagy. Shikonin has also been shown to inhibit liver cancer by causing mitochondrial dysfunction and increasing the oxidative stress level in tumor cells [8]. Apart from it antitumor activity, shikonin has been shown to have therapeutic effects against HIV infection, psoriasis, and other autoimmune diseases, but their underlying mechanisms are still unclear. Our previous research revealed that the PKM2 protein levels were elevated in the mDCs of SAA patients and that mDC activation is associated with increased intracellular PKM2 level. PKM2 regulates the immune status of SAA patients by enhancing the functions of mDCs and downstream cytotoxic T lymphocytes (CTLs) to prevent the aggravation of immune imbalance [9]. Hence, in this study, we focused on investigating the effect of shikonin on the immune status of SAA.

This study used an aplastic anemia (AA) mouse model obtained by inducing an autoimmune attack with total body irradiation (TBI) plus lymphocyte infusion. The AA mice were then treated with shikonin or CsA, and the results showed that shikonin affected the function of mDCs and CTLs *in vivo*. In addition, the high survival rates and blood counts in the AA mice were found to be associated with the shikonin-mediated downregulation of PKM2 levels.

## 2. Materials and Methods

### 2.1. Study Subjects

#### 2.1.1. Animals

The AA mouse models were obtained from Beijing Weitong Lihua Experimental Animal Technology Co. Ltd. To generate these AA mice, the first filial generation of inbred C57BL/6 mice and Balb/c mice was used as recipient mice (CB6F1, strain code: 303, 8 weeks old, SPF). These mice were major histocompatibility complex- (MHC-) heterozygous hybrids carrying H2^b/d^ with a high complement level. Allogeneic donor lymphocytes were obtained from C57BL/6 mice (H2^b/b^). Homologous wild-type C57BL/6 mice (strain code: 213, 8 weeks old, SPF) were used as the control mice. After the infusion of donor lymphocytes with MHC mismatch, T lymphocyte immune-mediated bone marrow destruction occurred in the CB6F1 mice, with pathogenesis mimicking that of human AA.

The obtained mice were bred and maintained at the Animal Center, Institute of Radiation Medicine, Chinese Academy of Medical Sciences. All animal-related studies were approved by the Tianjin Medical University intramural animal care and use committee.

#### 2.1.2. Induction of Bone Marrow Failure

Axillary and inguinal lymph nodes (LNs) were collected from C57BL/6 donors and then ground into homogenates, washed, centrifuged, and filtered. Their cell number was counted and adjusted to 10^7^/100 *μ*L.

The mice were divided into three groups: a normal control group (NC, *n* = 10), a TBI group (*n* = 10), and an AA model group (*n* = 17). The NC group contained CB6F1 mice. The TBI mice were given 4 Gy TBI using a cesium-137 gamma source. The AA model mice were also preirradiated with 4 Gy TBI using the same cesium-137 gamma source. After 4–6 h, diluted LN cells were injected through the retroorbital sinus into the recipient AA group mice (CB6F1) at 10 × 10^6^ cells/recipient in a 100 *μ*L lymphocyte separation solution (Solarbio, Beijing, China).

#### 2.1.3. Administration of Immunosuppressive Agents to the AA Mice

We divided the CB6F1 mice into three groups: CsA, shikonin, and normal saline (NS) groups. After infusion with donor lymphocytes for 1 h, the three groups were administered CsA (50 *μ*g/g/d, *n* = 10), shikonin (Sigma-Aldrich, USA) (100 *μ*g/g/d, *n* = 10), or normal saline (NS, *n* = 10), respectively, through the peritoneal cavity for 10 days. All mice were fed a healthy diet and then bled on the 17th day (see the specific operational steps in [Supplementary-material supplementary-material-1]).

### 2.2. Blood Counts and Immune Index

#### 2.2.1. Blood Counts

Blood samples were collected from the retroorbital sinuses of mice and anticoagulated with EDTA. Blood counts were determined using an automatic blood cell analyzer (MEK-722, Nihon Kohden).

#### 2.2.2. Flow Cytometry

Peripheral blood samples obtained by the above-described method were incubated with red blood cell lysate solution (BD Biosciences, USA) for 20 min to lyse the red blood cells (RBCs). The lysates were stained with antibodies and analyzed using a flow cytometer (FACSCalibur, BD Biosciences, USA).

Monoclonal antibodies against murine CD3, CD4, CD8, CD11c, CD80, CD86, Foxp3, PKM2, CD25, MHC II, CD107a, and CD107b were obtained from BD Biosciences (San Diego, CA, USA).

#### 2.2.3. Determination of Glucose Consumption, Pyruvate Generation, and ATP Content

After eyeball extraction, the peripheral blood samples of the mice were collected and anticoagulated with EDTA. The mDCs were sorted and counted using a mouse mDC immunomagnetic sorting kit (Miltenyi Biotec, Germany). The supernatant of the cell culture was used to measure the amount of glucose consumption using a Glucose Assay Kit (Nanjing Institute of Biological Engineering, Nanjing, China) according to the kit's instructions. The pyruvate and ATP concentrations in the cells were measured using a Pyruvate Assay Kit (Nanjing Institute of Biological Engineering, Nanjing, China) and an ATP Assay Kit (Beyotime Biotechnology, Shanghai, China), respectively, according to the kit instructions. The optical densities at 505 nm for glucose measurement and pyruvate generation, and at 636 nm for ATP content, were read using an ELISA reader (BioTek Instruments, Inc., USA).

#### 2.2.4. Quantitative RT-PCR (qRT-PCR)

The CD8+ T cells from the mice were sorted by immunomagnetic separation. Total RNA was extracted and subjected to reverse transcription.

qRT-PCR was performed in an iQ5 PCR system (Bio-Rad, Hercules, CA, USA) under the following thermal cycling conditions: initial denaturation at 95°C for 2 min, followed by 40 cycles of amplification (95°C for 10 s, indicated annealing temperature for 35 s, 72°C for 30 s, and 65°C for 10 s). The primer sequences were as follows: Perforin, F: 5′-CAGGTCAACATAGGCATGCACG-3′ and R: 5′-GAACAGCAGGACGTTAATGGAG-3′; Granzyme B, F: 5′-GAAACGCTACTAACTACAGG-3′ and R: 5′-CCACTGAGCTAAGAGGT-3′; and *β*-actin, F: 5′-TTGCCGACACGATGCAGAA-3′ and R: 5′- GCCGATCCACACCGTGTACT-3′. The relative expression levels of genes of interest were calculated.

#### 2.2.5. Survival Rates

The survival rates of the mice from the NC (*n* = 10), TBI (*n* = 10), AA model (*n* = 5), shikonin (*n* = 5), CsA (*n* = 5), and NS (*n* = 5) groups for 90 days or until death were calculated and compared. Blood counts were determined at days 17, 30, and 90 after the induction of AA.

#### 2.2.6. Statistical Analysis

Data are presented as mean ± standard deviation. Statistical analyses were performed using the chi-square test, followed by the nonparametric unpaired *t*-test. *p* values < 0.05 were considered to indicate significance. One-way analysis of variance was performed for the comparison of three independent groups.

## 3. Results

### 3.1. Blood Counts Detected in NC, TBI, and AA Model Groups

**Figure 1:**
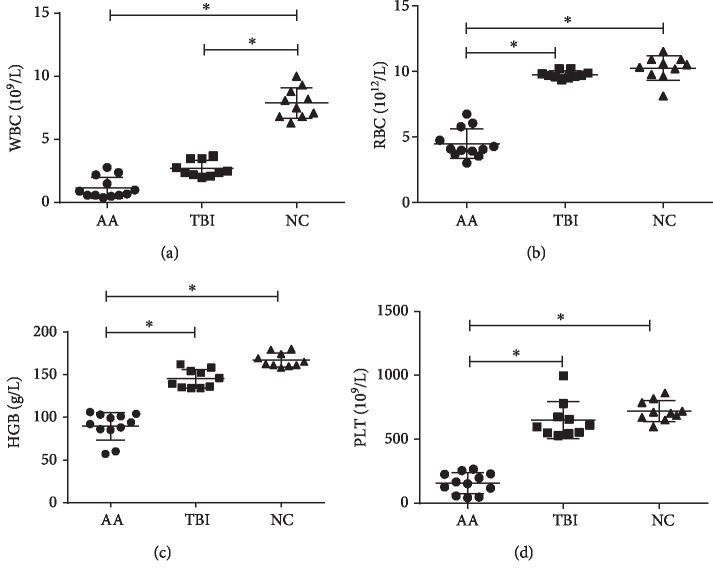
The peripheral blood tests of the AA group CB6F1 mice showed aleukocytosis (a), anemia (b, c), and thrombocytopenia (d) compared with the NC group and TBI group (^∗^*p* < 0.01). AA: aplastic anemia (*n* = 12); TBI: total body irradiation (*n* = 10); NC: normal control (*n* = 10).

The counts of white blood cells (WBCs), RBCs, hemoglobin (Hb), and platelets (PLTs) in the peripheral blood samples of the CB6F1 mice were 7.89 ± 1.21 × 10^9^/L, 10.24 ± 0.93 × 10^12^/L, 166.9 ± 8.14 g/L, and 720.3 ± 80.39 × 10^9^/L, respectively, in the NC group; 2.71 ± 0.63 × 10^9^/L, 9.74 ± 0.28 × 10^12^/L, 145 ± 10.79 g/L, and 648.4 ± 144.2 × 10^9^/L, respectively, in the TBI group; and 1.18 ± 0.84 × 10^9^/L, 4.47 ± 1.13 × 10^12^/L, 89.58 ± 16.17 g/L, and 156.1 ± 80.29 × 10^9^/L, respectively, in the AA model group. The peripheral blood tests of the AA model group showed pancytopenia compared with those of the NC and TBI groups (Table 1, Figure 1) (*p* < 0.0001).

### 3.2. PKM2+ mDCs Increased in the AA Mice and Decreased after Shikonin Treatment

**Figure 2:**
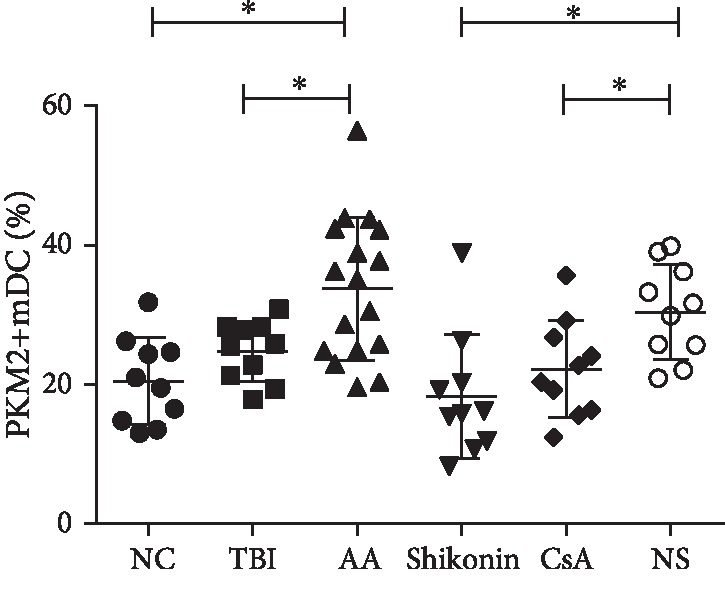
The PKM2+ mDC proportion of mDCs (PKM2+CD11c+/CD11c+) in the AA group was higher than those of the NC and TBI groups (*n* = 10, *p* < 0.05). There was no significant difference between the NC and TBI groups (*n* = 10, *p* > 0.05). The PKM2 level is decreased in mDCs of the shikonin and CsA groups compared with the NS group (*n* = 10, *p* < 0.05). There was no significant difference between the shikonin and CsA groups (*p* > 0.05). AA: aplastic anemia (*n* = 17); TBI: total body irradiation (*n* = 10); NC: normal control (*n* = 10); shikonin (*n* = 10); CsA: cyclosporin A (*n* = 10); NS: normal saline (*n* = 10) (^∗^*p* < 0.05).

The proportion of PKM2+ mDCs (PKM2+CD11c+/CD11c+) was higher in the AA group (33.76 ± 10.21%) compared with those in the NC group (20.54 ± 6.22%) and the TBI group (24.81 ± 4.28%) (*p* < 0.05). The proportion was not significantly different between the NC and TBI groups (*p* > 0.05). After the treatments, the PKM2 levels in the mDCs decreased in the shikonin (18.33% ± 2.81%, *n* = 10) and CsA (22.25 ± 2.21%, *n* = 10) groups compared with that in the NS group (30.44 ± 2.14%, *n* = 10) (*p* < 0.05). The levels were not significantly different between the shikonin and CsA groups (*p* > 0.05) (Figure 2).

### 3.3. The Expression of mDC Costimulatory Molecules CD80 and CD86 Increased in the AA Mice and That of CD86 Decreased after Shikonin Treatment

To verify whether PKM2 overexpression was responsible for the activation of mDCs, the expressions of costimulatory molecules CD86 and CD80 on the mDCs were evaluated. The percentages of mDCs in the peripheral blood samples of NC, TBI, AA, shikonin, CsA, and NS groups were 12.32 ± 2.61%, 14.02 ± 3.73%, 14.64 ± 4.67%, 15.09 ± 2.90%, 13.36 ± 4.48%, and 14.92 ± 3.53%, respectively, indicating that the differences between the six groups were not significant (*p* > 0.05, [Supplementary-material supplementary-material-1]).

**Figure 3:**
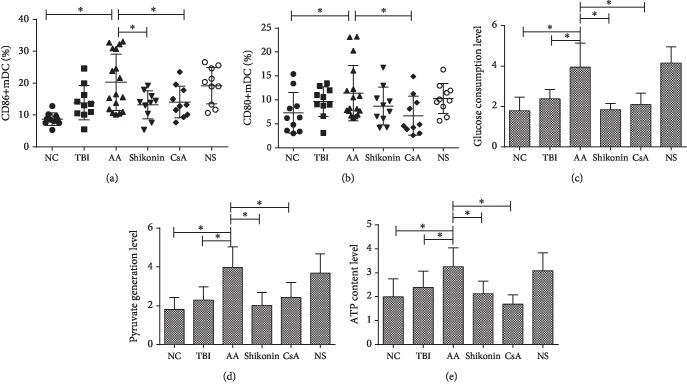
(a) The CD86+ mDCs in the AA group significantly increased compared with those of the NC and TBI groups (*p* < 0.05). After the treatments, the CD86 levels in the shikonin and CsA groups decreased significantly compared with that in the AA group (*p* < 0.05). (b) The CD80+ mDCs in the AA group significantly increased compared with those in the NC group (*p* < 0.05). The CD80 levels in the CsA group decreased compared with those in the AA group (*p* < 0.05). (c) The glucose consumption levels in mDCs of the AA group were significantly higher than those in the TBI and NC groups, while those in the shikonin and CsA groups were significantly lower than those in the AA group (*p* < 0.05). (d) The pyruvate levels in mDCs of the AA group were significantly higher than those in the TBI and NC groups, while those in the shikonin and CsA groups were significantly lower than those in the AA group (*p* < 0.05). (e) The ATP contents in mDCs in the AA group were significantly higher than those in the TBI and NC groups, while those in the shikonin and CsA groups were significantly lower than those in the AA group (*p* < 0.05). AA: aplastic anemia (*n* = 17); TBI: total body irradiation (*n* = 10); NC: normal control (*n* = 10); shikonin (*n* = 10); CsA: cyclosporin A (*n* = 10); NS: normal saline (*n* = 10) (^∗^*p* < 0.05).

The proportions of CD86+ mDCs in the NC, TBI, AA, shikonin, CsA, and NS groups were 8.68 ± 1.96%, 13.87 ± 5.39%, 20.27 ± 8.41%, 13.21 ± 4.35%, 14.06 ± 4.96%, and 19.21 ± 5.73%, respectively. Notably, the proportion was significantly higher in the AA group than those in the NC and TBI groups (*p* < 0.05). After the treatments, the CD86 expression in the shikonin and CsA groups decreased significantly compared with that in the AA group (*p* < 0.05, Figure 3(a)).

The percentages of CD80+ mDCs in the NC, TBI, AA, shikonin, CsA, and NS groups were 7.3 ± 4.24%, 9.65 ± 3.11%, 11.42 ± 5.77%, 8.72 ± 3.95%, 6.69 ± 4.08%, and 10.28 ± 3.13%, respectively. The percentage of CD80+ mDCs in the AA group was elevated compared with that in the NC group (*p* < 0.05). After the treatments, the CD80 expression in the CsA group decreased significantly compared with that in the AA group (*p* < 0.05). However, no statistically significant difference was observed in the CD80 expression between the shikonin and AA groups (Figure 3(b)).

### 3.4. The Glucose Consumption Level, Pyruvate Level, and ATP Content in mDCs Increased in the AA Mice and Decreased after Shikonin Treatment

We measured the glucose consumption level, pyruvate generation level, and ATP content to determine the level of glycolytic metabolism in the mDCs of each group. The glucose consumption levels of mDCs in the NC, TBI, AA, shikonin, CsA, and NS groups were 1.79 ± 0.67, 2.38 ± 0.47, 3.94 ± 1.19, 1.83 ± 0.31, 2.11 ± 0.55, and 4.15 ± 0.81, respectively. Notably, the level in the AA group was significantly higher than those in the TBI and NC groups (*p* < 0.05). After the treatments, the glucose consumption levels of mDCs in the shikonin and CsA groups reduced significantly compared with that in the AA group (*p* < 0.05, Figure 3(c)).

The pyruvate generation levels of mDCs in the NC, TBI, AA, shikonin, CsA, and NS groups were 1.80 ± 0.62, 2.29 ± 0.69, 3.97 ± 1.07, 2.02 ± 0.66, 2.43 ± 0.77, and 3.68 ± 1.00, respectively. Notably, the level in the AA group was significantly higher than those in the TBI and NC groups (*p* < 0.05). After the treatments, the pyruvate generation levels of mDCs in the shikonin and CsA groups reduced significantly compared with that in the AA group (*p* < 0.05, Figure 3(d)).

The ATP contents of mDCs in the NC, TBI, AA, shikonin, CsA, and NS groups were 1.99 ± 0.75, 2.38 ± 0.68, 3.25 ± 0.79, 2.12 ± 0.53, 1.68 ± 0.40, and 3.08 ± 0.75, respectively. Notably, the ATP content in the AA group was significantly higher than those in the TBI and NC groups (*p* < 0.05). After the treatments, the ATP contents of mDCs in the shikonin and CsA groups decreased significantly compared with that in the AA group (*p* < 0.05, Figure 3(e)).

### 3.5. The mRNA Expression of Granzyme B in CD8+ Cells Increased in the AA Mice and Decreased after Shikonin Treatment

**Figure 4:**
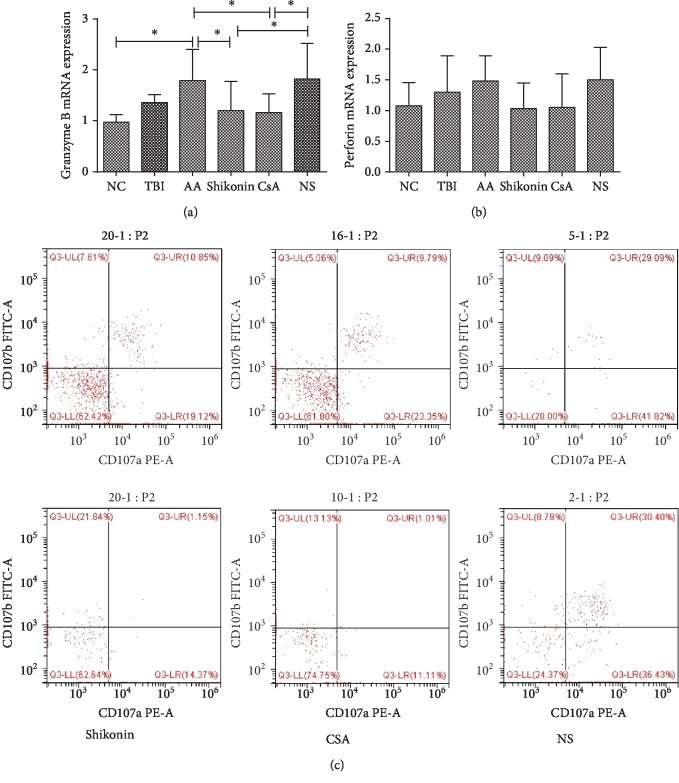
(a) The granzyme B mRNA expression of CD8+ cells in the AA group was significantly higher than that in the NC group, while those in the shikonin and CsA groups were significantly decreased compared with the AA and NS groups (*p* < 0.05). (b) The level of perforin mRNA expression in CD8+ cells did not show a statistically significant difference among the six groups (*p* > 0.05). (c) The ratio of CD107a+CD8+/CD8+ cells and CD107b+CD8+/CD8+ cells in the AA group was increased compared with that in the NC group, while those in the shikonin and CsA groups were significantly decreased compared with that in the NS group (*p* < 0.05). AA: aplastic anemia (*n* = 17); TBI: total body irradiation (*n* = 10); NC: normal control (*n* = 10); shikonin (*n* = 10); CsA: cyclosporin A (*n* = 10); NS: normal saline (*n* = 10).

The granzyme B mRNA expression in the CD8+ cells of the AA group (1.79 ± 0.61) was significantly higher than that in the CD8+ cells of the NC group (0.97 ± 0.48, *p* < 0.05). Notably, the expressions in the shikonin (1.20 ± 0.57) and CsA (1.16 ± 0.37) groups was lower than those in the AA and NS groups (1.82 ± 0.70) (*p* < 0.05, Figure 4(a)). However, the perforin mRNA expression in the CD8+ cells showed no significant difference between the six groups (*p* > 0.05, Figure 4(b)).

We further investigated the degranulation levels of CTLs. The ratio of CD107a+CD8+/CD8+ cells (expressed as a percentage) was significantly higher in the AA group (72.4 ± 7.41%) compared with that in the NC group (20.73 ± 3.71%) (*p* < 0.05) but showed no significant difference between the NC and TBI groups (*p* > 0.05). After the treatments, the CD107a levels in the CD8+ cells decreased significantly in the shikonin (45.08 ± 5.96%) and CsA (33.56 ± 5.74%) groups compared with that in the NS group (71.65 ± 7.33%) (*p* < 0.05). Notably, the difference in the CD107a levels between the shikonin and CsA groups was not significantly different (*p* > 0.05). The ratios of CD107b+CD8+/CD8+ cells (expressed as a percentage) in the NC, TBI, AA, shikonin, CsA, and NS groups were 14.94 ± 1.44%, 23.03 ± 6.1%, 45.54 ± 8.56%, 19.39 ± 2.43%, 25.88 ± 2.12%, and 41.54 ± 3.99%, respectively. The ratio in the AA group was significantly higher than that in the NC group (*p* < 0.05). After the treatments, the ratio in the shikonin and CsA groups reduced significantly compared with that in the AA group (*p* < 0.05, Figure 4(c)).

### 3.6. The Influence of PKM2 Inhibition on Other Immune Cells

**Figure 5:**
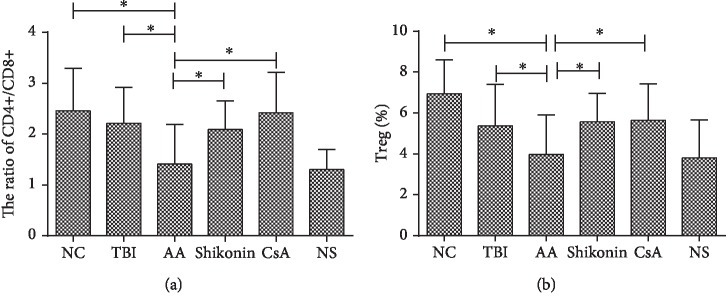
(a) The ratio of CD4+/CD8+ in the AA group was significantly lower than those in the TBI, NC, shikonin, and CsA groups (*p* < 0.05). (b) The frequency of Treg in the AA group was significantly lower than those in the TBI, NC, shikonin, and CsA groups (*p* < 0.05). AA: aplastic anemia (*n* = 17); TBI: total body irradiation (*n* = 10); NC: normal control (*n* = 10); shikonin (*n* = 10); CsA: cyclosporin A (*n* = 10); NS: normal saline (*n* = 10) (^∗^*p* < 0.05).

We also explored the effects of PKM2 on other immune cells in addition to mDCs. The ratio of CD4+/CD8+ cells in the AA group (1.41 ± 0.78) was significantly lower than those in the TBI (2.21 ± 0.71, *p* < 0.05), NC (2.46 ± 0.84, *p* < 0.05), shikonin (2.09 ± 0.57, *p* < 0.05), and CsA (2.42 ± 0.80, *p* < 0.05) groups (Figure 5(a)).

The ratio of CD4+CD25+Foxp3+ cells (Treg) in the AA group (3.96 ± 1.94) was significantly lower than those in the TBI (5.36 ± 2.04, *p* < 0.05), NC (6.94 ± 1.66, *p* < 0.05), shikonin (5.57 ± 1.39, *p* < 0.05), and CsA (5.64 ± 1.80, *p* < 0.05) groups (Figure 5(b)).

### 3.7. The Effect of Shikonin on the Survival Rates of the AA Mice

**Figure 6:**
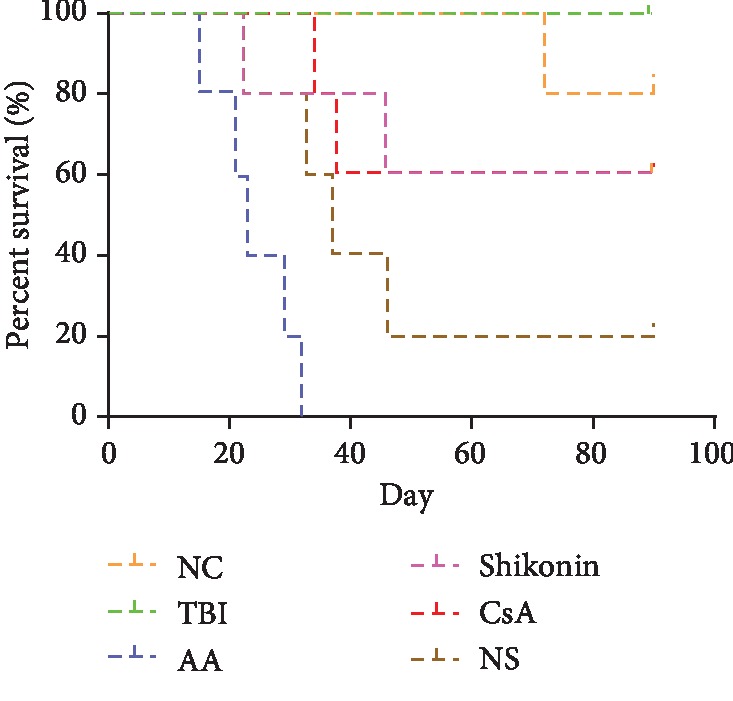
The survival rates of the mice from the NC (*n* = 10), TBI (*n* = 10), AA model (*n* = 5), shikonin (*n* = 5), CsA (*n* = 5), and NS (*n* = 5) groups were examined and compared for 90 days or until death. The mice from the Shikonin and CsA groups had a higher survival rate than the AA model and NS groups (*p* < 0.05). No significant difference was observed between the TBI and NC groups. AA: aplastic anemia; TBI: total body irradiation; NC: normal control; CsA: cyclosporin A; NS: normal saline.

Mice from the NC, TBI, AA model, shikonin, CsA, and NS groups were housed to evaluate their 90-day survival rates. The findings revealed that 90% (9/10) of the NC group, 100% (10/10) of the TBI group, 0% (0/5) of the AA model group, 60% (3/5) of the shikonin group, 60% (3/5) of the CsA group, and 20% (1/5) of the NS group survived to day 90. At day 46, 60% of the mice from the shikonin and CsA groups were alive, and all survived until they were euthanized at the end of the experiment, but only one mouse in the NS group survived to day 46. Meanwhile, none of the mice in the AA model group survived till day 32. As shown in Figure 6, 90 days after the first administration of shikonin or CsA, the survival rates in the shikonin and CsA groups were higher than those in the AA model and NS groups (*p* < 0.05). The survival rates were not significantly different between the TBI and NC groups.

**Figure 7:**
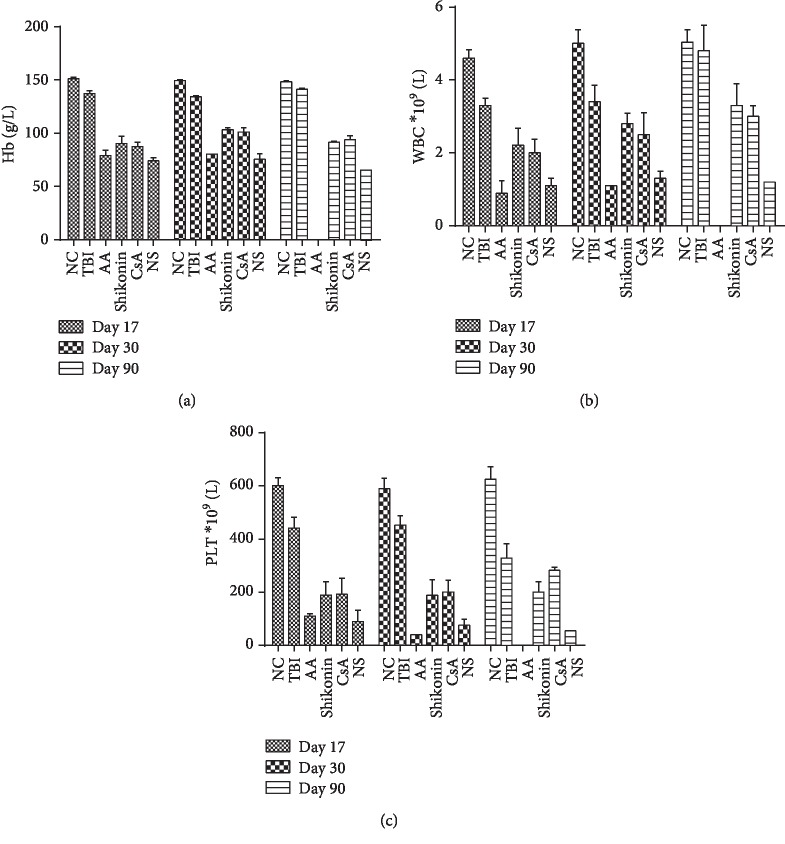
Mice peripheral blood counts at day 17, day 30, and day 90. Until the time of death, no improvement was observed in (a) Hb, (b) WBC, and (c) PLT in the mice from the AA model and NS groups, whereas the blood counts from the shikonin and CsA groups gradually recovered over time, especially leukocytes (*p* < 0.05). AA: aplastic anemia (*n* = 17); TBI: total body irradiation (*n* = 10); NC: normal control (*n* = 10); shikonin (*n* = 10); CsA: cyclosporin A (*n* = 10); NS: normal saline (*n* = 10).

The blood counts of the mice from the TBI group reached close to normal levels by day 90. The TBI plus lymphocyte infusion led to unrecoverable reduction in the WBC counts. Until the time of death, the blood counts of the mice in the AA model and NS groups showed no improvement, whereas those of the mice in the shikonin and CsA groups, especially leukocyte counts, gradually recovered.  Figure 7 shows the WBC, Hb, and PLT counts in mice from different groups.

## 4. Discussion

SAA is a hematological disease characterized by bone marrow hematopoietic failure. Although its etiology and pathogenesis are complex, recent data suggest that AA is an immune-mediated disease [1]. According to recent research, dysfunctional mDCs play a role in SAA pathogenesis [3]. However, the mechanisms of mDC activation and of the resulting induction of a series of autoimmune damage processes are still unclear.

Shikonin is a major active chemical component extracted from the traditional Chinese medicinal herb *L*. *erythrorhizon*. Studies have proven that shikonin is a highly specific PKM2 inhibitor and plays an important role in suppressing breast cancer, hepatocellular carcinoma, and other tumors. In recent years, research on the roles of shikonin in human health has gradually deepened. Accumulating evidence suggests that shikonin can suppress tumor growth and overcome the resistance to chemotherapy drugs in tumor cells [10]. Tang et al. [11] suggested that shikonin can significantly inhibit the proliferation of esophageal cancer cells by regulating the HIF1*α*/PKM2 signaling pathway. Shikonin can also suppress the inflammatory immune response by blocking inflammatory signals, thereby delaying the progression of inflammatory diseases such as sepsis. Furthermore, it has been reported to inhibit T lymphocyte activation by inhibiting IKK*β* activity and JNK signaling and is thus expected to play a therapeutic role as a potential immunosuppressant in autoimmune diseases [12]. Given the extensive therapeutic effects of shikonin against immunological diseases, inflammation, and tumors, in this study, we attempted to investigate the therapeutic role of shikonin against SAA pathogenesis.

DCs are the only antigen-presenting cells that can activate naïve T cells. They can be divided into several subsets based on their origin, location, and function. mDCs play a unique role in the generation of T lymphocyte-mediated immune responses [13]. The ability of mDCs to activate T cells depends on their maturation and functional differentiation status and is also affected by environmental factors such as microbial products, cytokines, oxygenase, metabolites, and immunosuppressive agents [14, 15]. mDCs can regulate the function and proliferation of T cells and promote the activation of Th1 and CD8+ T cells in immune responses. mDCs also play a role in the regulation of Treg differentiation by modulating the expression of programmed cell death ligand 1 and T cell costimulatory molecules [16]. In mice, monocytes can differentiate to mDCs under inflammatory conditions *in vivo* or *in vitro* after stimulation with granulocyte macrophage colony-stimulating factor and interleukin 4 [17]. mDCs highly express CD11c, MHC-II, and DC-specific transcription factors [18]. Our previous study showed that the mDCs of newly diagnosed SAA patients exhibit elevated PKM2 protein levels and that this protein is involved in the activation of mDC function [8].

In this study, shikonin and CsA were used to treat AA model mice to observe their effect on the immune status of these mice. CB6F1 mice were used to generate AA mouse models of bone marrow failure by inducing autoimmune attack. The parental lines of the CB6F1 mice were Balb/C mice (H2^d/d^) and C57BL/6 mice (H2^b/b^). Because CB6F1 mice are MHC-heterozygous hybrids carrying H2^b/d^, they could easily initiate autoimmune reaction with high complement level. In a previous study, a lethal dose of irradiation was found to damage the hematopoietic tissue of the bone marrow microenvironment in CB6F1 mice. At this point, the C57BL/6 mouse (H2^b/b^) lymphocytes, which are MHC-mismatched, could induce sustained hematopoietic impairment of the immune bone marrow [19]. In our study, the SAA model mice showed a decrease in blood counts. A decreased ratio of CD4+/CD8+ cells, increased expressions of CD80 and CD86 on mDCs, increased levels of CTL degranulation, and inadequate Treg number were also detected. The state of immune dysfunction in these AA mice was similar to that in SAA patients. We found that the proportion of PKM2+ mDCs in the AA mice was elevated and was significantly positively correlated to the function of mDCs and CTLs. PKM2 activation in mDCs was confirmed in *in vivo* mice experiments, but the PKM2 levels in mDCs decreased after shikonin or CsA treatment. The expression of mDC co-stimulatory molecules CD80 and CD86 increased in the AA mice and that of CD86 decreased after shikonin treatment. This finding suggests that shikonin reduced the expression of mDC costimulatory molecules by reducing PKM2 levels, which inhibited mDC activation, rather than reducing the number of mDCs. The glucose consumption level, pyruvate generation level, and ATP content were also increased in the mDCs of the AA mice. This finding suggests that shikonin blocked cell glycolytic metabolism to some extent, thereby inhibiting the activation and proliferation of mDCs. The level of glycolysis in the mDCs also decreased after shikonin and CsA treatments.

During antigen presentation, mDCs gradually mature and express high levels of the costimulatory molecules CD80 and CD86 [20]. CTLs are activated through the combination of mDCs and numerous cellular signals, which initiates an immune response. As the main effector cells with cytotoxic function CTLs are closely related to SAA pathogenesis, so we also explored the effect of shikonin on CTLs in the AA mice. Our data showed that shikonin decreased the level of the cytotoxic molecule granzyme B in CD8+ T cells and could regulate the ratio of CD4+/CD8+ cells and the Treg count in the AA mice. The effects of shikonin were similar to those of CsA. The expression of CD107a/b on the surface of CD8+ T cells in the AA group increased relative to that in the controls but reduced after shikonin or CsA administration. CD8+ T cells store most of the cytotoxic molecules in cytotoxic granules, which degranulate upon contact with the target cell. As a lysosomal-associated membrane glycoprotein, CD107a/b is expressed on the surface of CD8+ T cells [21]. When CD8+ T cells come in contact with target cells, the costimulatory molecules fuse with the target cell membrane; this causes the release of the cytotoxic molecules from the T cell granules, eventually leading to the death of the target cells [22]. The upregulation of CD107a/b expression on CTLs observed in our study was consistent with the increase in granzyme B secretion. Thus, the results above indicate that the CTL activity in the AA group was significantly increased relative to that in the controls but reduced after administering shikonin or CsA. We speculate that the changes in CTL function were due to shikonin-mediated inhibition of PKM2 levels in mDCs, which in turn regulated the expression of mDC costimulatory molecules and inflammatory factors. Recently, the role of Treg cell population in SAA pathogenesis has attracted much research attention [10]. Tregs are believed to control the progression of autoimmunity in AA by suppressing overactive T cells and preventing the antigen presentation process of mDCs [23]. Our data showed inadequate numbers of Tregs in the AA mice. When treated with shikonin or CsA, the number of Tregs increased, which is consistent with the insufficient Tregs found in human AA that cannot inhibit the autologous effector T cells, eventually leading to T cell-mediated bone marrow failure. After shikonin treatment reduced the activity of mDCs, the inhibitory effect of mDCs on Tregs also decreased.

Additionally, we evaluated the survival rates and peripheral blood counts of mice at different time points to explore the effects of shikonin and the immunosuppressant CsA. Our SAA mouse model generated by the combined use of TBI plus lymphocyte infusion exhibited a mortality rate of almost 100% within 32 days. However, treatment with shikonin or CsA reduced the mortality rate to 40%. The survival rate increased and PKM2 expression decreased after shikonin administration in the shikonin group compared with those in the AA model and NS groups. The finding demonstrated that the high survival rates in mice treated with shikonin were associated with shikonin-induced inhibition of PKM2 expression. After a short period of bone marrow suppression, the blood counts of the TBI group quickly returned to the normal levels. However, the blood counts of the AA model and NS groups showed no improvement until the time of death, whereas those, especially leukocyte counts, of the shikonin and CsA groups gradually recovered. This finding indicates that the mice experienced partial remission in blood counts after shikonin or CsA treatment compared with the AA model and NS groups. This result is consistent with our previous finding in *in vitro* studies using human cell lines that the PKM2 expression level in the mDCs is significantly associated with SAA progression [9]. Shikonin administration in AA mice was conducive to disease recovery, which again confirmed the therapeutic significance of shikonin against SAA pathogenesis.

In summary, shikonin could inhibit the activation and proliferation of mDCs and inhibit the activation of their downstream immune effector cells by reducing PKM2 levels in mDCs. Thus, shikonin is a potential new drug target for SAA immunotherapy.

## Figures and Tables

**Table 1:** Blood counts in the NC, TBI, and AA model groups.

	NC group (*n* = 12)	TBI group (*n* = 10)	AA group (*n* = 10)
WBC (×10^9^/L)	7.89 ± 1.21^∗^	2.71 ± 0.63^#^	1.18 ± 0.84^∗#^
NEU (×10^9^/L)	0.52 ± 0.11^∗^	0.38 ± 0.15^#^	0.24 ± 0.11^∗#^
LYM (×10^9^/L)	6.67 ± 0.56^∗^	1.97 ± 0.37^#^	0.79 ± 0.25^∗#^
RBC (×10^12^/L)	10.24 ± 0.93^∗^	9.74 ± 0.28^#^	4.47 ± 1.13^∗#^
HGB (g/L)	166.9 ± 8.14^∗^	145 ± 10.79^#^	89.58 ± 16.17^∗#^
PLT (×10^9^/L)	720.3 ± 80.39^∗^	648.4 ± 144.2^#^	156.1 ± 80.29^∗#^

^∗^The peripheral blood tests of the AA group showed pancytopenia compared with the NC group (*p* < 0.0001). ^#^The peripheral blood tests of the AA group showed pancytopenia compared with the TBI group (*p* < 0.0001).

## Data Availability

The data used to support the findings of this study are available from the corresponding author upon request.

## Supplementary Materials

Supplementary Materials Figure S1: the percentage of mDCs in the peripheral blood of mice in the NC, TBI, AA, PKM2-i, CsA, and NS groups were tested by FCM. Among mononuclear cells, mDCs were identified as CD11c+MHC II+. There was no significant difference among the six groups. (*p* > 0.05). Table S1: specific operational steps in mice.
