# Supplementary material for: Effects of Shikonin on the Functions of Myeloid Dendritic Cells in a Mouse Model of Severe Aplastic Anemia
Source: Mediators Inflamm. 2020 Feb 20;2020:9025705. doi: 10.1155/2020/9025705 (PMC7053458; doi:10.1155/2020/9025705)
Supplement: Supplementary Materials — Figure S1: the percentage of mDCs in the peripheral blood of mice in the NC, TBI, AA, PKM2-i, CsA, and NS groups were tested by FCM. Among mononuclear cells, mDCs were identified as CD11c+MHC II+. There was no significant difference among the six groups. (p > 0.05). Table S1: specific operational steps in mice. [file 9025705.f1.docx]

**Supplementary Figure legends**

Figure S1. The percentage of mDCs in theperipheral blood of the mice in the NC, TBI, AA, PKM2-i, CsA and NS groups were test by FCM. Among mononuclear cells, mDCs were identified as CD11c+MHC II+. There was no significant difference among the six groups. (P>0.05)

**Supplementary Figures**

Figure S1


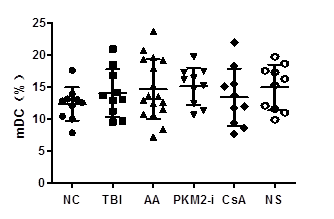


**Supplementary tables**

**Table S1.** Specific operational steps in the mice

| **Group** | **Day 0**  **(0 hour)** | **Day 0**  **(4-6 hour)** | **Day 0**  **(1 hour after LN infusion) ~ Day 9** | **Day 17** | **Day 30**  **(or until death)** | **Day 90**  **(or until death)** |
| --- | --- | --- | --- | --- | --- | --- |
| NC |  |  |  | Bleeding | Bleeding | Bleeding |
| TBI | TBI |  |  | Bleeding | Bleeding | Bleeding |
| AA module | TBI | LN infusion |  | Bleeding | Bleeding | Bleeding |
| PKM2-i | TBI | LN infusion | shikonin (100µg/g/d) | Bleeding | Bleeding | Bleeding |
| CsA | TBI | LN infusion | CsA(50µg/g/d) | Bleeding | Bleeding | Bleeding |
| NS | TBI | LN infusion | NS | Bleeding | Bleeding | Bleeding |

NC : normal control ; NS: normal saline.
